# Mapping Menstrual and Pelvic Health Scenario in India: A Scoping Review of Biopsychosocial Factors

**DOI:** 10.7759/cureus.85541

**Published:** 2025-06-07

**Authors:** Suroshree Mitra, Anu Arora, Apurv Shimpi

**Affiliations:** 1 Department of Physiotherapy, School of Physiotherapy, D. Y. Patil (Deemed to be) University, Navi Mumbai, IND; 2 Department of Physiotherapy, Sancheti Institute for Orthopedics and Rehabilitation College of Physiotherapy, Maharashtra University of Health Sciences, Pune, IND

**Keywords:** adolescent girls, biopsychosocial model, menstrual health, menstrual hygiene management, pelvic health

## Abstract

Menstrual and pelvic health are critical for adolescent girls’ well-being, yet cultural taboos, inadequate education, and limited resources in India hinder effective menstrual hygiene management (MHM) and contribute to adverse health and social outcomes. This scoping review maps the knowledge, attitudes, and practices related to pelvic health, menstrual health, and hygiene among adolescent girls in India, applying the biopsychosocial model to identify gaps and inform interventions. Following the guidelines, a comprehensive search was performed on PubMed and ScienceDirect using key terms (“menstrual health” OR “menstrual hygiene”) AND (“pelvic health”) AND (“adolescents” OR “school girls”) AND (“India”). Based on the eligibility criteria, a total of seven studies were included, which revealed pervasive myths (e.g., menstrual impurity), limited premenarche knowledge (23-81.8% awareness), and inadequate MHM practices (e.g., 63% rural cloth use). Negative attitudes, driven by taboos, caused shame and school absenteeism (17-24%). Poor water, sanitation, and hygiene facilities and nonstandardized curricula exacerbated gaps. Government initiatives lack uniform implementation, and cultural, educational, and environmental barriers undermine adolescent menstrual health in India. Standardized, gender-inclusive education, improved facilities, and culturally sensitive interventions are needed to enhance MHM and reduce health disparities, fostering adolescent well-being.

## Introduction and background

Adolescence marks a critical period of physical, cognitive, and psychosocial transitions, with puberty playing a pivotal role in shaping lifelong health outcomes, particularly for girls [1]. Menstrual health and hygiene, encompassing the effective management of menstruation and pelvic health, are essential for adolescent well-being. Yet, they remain underexplored in low- and middle-income countries (LMICs) like India [2,3]. Pelvic health, defined as the optimal functioning and management of bladder, bowel, and reproductive organs, extends beyond the absence of disease, aligning with the World Health Organization’s holistic health framework [4,5]. However, cultural taboos, inadequate education, and limited access to resources create significant barriers to menstrual and pelvic health in India, contributing to adverse health and social outcomes [4,6].

Emerging evidence highlights a growing burden of noncommunicable diseases, including pelvic floor disorders, among young women, often linked to poor menstrual hygiene and lack of early education [7,8]. In India, adolescent girls face challenges such as menstrual pain, infections, and psychosocial distress (e.g., fear, shame, embarrassment), which disrupt school attendance and academic performance [9,10]. Government initiatives, such as the Menstrual Hygiene Scheme under the National Rural Health Mission, the Swachh Bharat Mission (2014), and the annual Menstrual Hygiene Day (May 28), aim to improve sanitation and awareness [11,12]. However, these programs often lack a standardized, holistic approach integrating physical, psychological, and hygiene domains, resulting in uneven knowledge and practice across communities [6,12].

Education about puberty, deemed “crucial” by the United Nations Educational, Scientific and Cultural Organization (UNESCO), is vital for empowering adolescents to navigate menstruation confidently [7]. Yet, school curricula in India frequently focus narrowly on biological aspects, neglecting menstrual hygiene, pelvic health, and psychosocial well-being [13,14]. Cultural stigma and gender disparities further limit open discussions, particularly involving boys, perpetuating myths and social taboos [15,16]. The biopsychosocial model offers a comprehensive framework to understand these dynamics, emphasizing the interplay of biological (e.g., menstrual pain), psychological (e.g., anxiety), and social (e.g., taboos) factors in shaping menstrual health experiences [9,10].

Despite the importance of menstrual health, systematic reviews on this topic in India are scarce, heterogeneous, and often of poor quality, with inconsistent outcome measures and high risks of bias [5,17]. Existing reviews focus predominantly on menstrual hygiene management (MHM) without adequately addressing pelvic health or the broader sociocultural and educational context [6,18]. The rationale for this scoping review stems from the need to consolidate fragmented evidence on adolescent menstrual and pelvic health in India, where cultural, educational, and environmental barriers persist. Unlike systematic reviews, which focus on quantitative synthesis to answer specific questions, scoping reviews aim to provide a comprehensive overview of existing literature, identifying key themes, gaps, and research priorities without requiring meta-analysis. This review includes pelvic health to capture its interplay with menstrual health, such as infection risks from poor hygiene, though the literature predominantly focuses on menstrual health due to limited studies on pelvic health outcomes in adolescents. By applying the biopsychosocial model, this review maps how biological, psychological, and social factors shape knowledge, attitudes, and practices (KAP) related to pelvic and menstrual health among adolescent girls in India, synthesizing evidence from diverse settings to identify sociocultural influences, assess gaps in knowledge and resources, evaluate environmental barriers, and recommend evidence-based strategies to enhance adolescent health and well-being.

## Review

The methodology for the present scoping review adhered to the Preferred Reporting Items for Systematic Reviews and Meta-Analyses extension for Scoping Reviews (PRISMA-ScR) and the framework of Arksey and O’Malley (2005) [19,20]. The methodology involved five stages, which are outlined below [21].

Stage 1: Identification of the research question

The purpose of this review was to map how biological, psychological, and social factors influence KAP related to pelvic and menstrual health among adolescent girls in India. The primary research question framed for the review was “How do biological, psychological, and social factors shape the KAP related to pelvic and menstrual health among adolescent girls in India?” The secondary objectives explored were as follows: (1) What are the sociocultural factors influencing pelvic and menstrual health and hygiene? (2) What are the gaps in access to knowledge and resources for pelvic and menstrual health management? (3) What environmental and educational barriers affect menstrual hygiene practices? (4) What recommendations can be made to improve awareness and education on pelvic and menstrual health in India?

Stage 2: Identification of relevant studies

An extensive literature search was conducted using the databases PubMed and ScienceDirect. The search was limited to studies published in English between 2011 and 2022. A combination of Medical Subject Headings (MeSH) terms and Boolean operators was used, including (“menstrual health” OR “menstrual hygiene”) AND (“pelvic health”) AND (“adolescents” OR “school girls”) AND (“India”). The search aimed to capture a wide range of studies that discuss knowledge, perceptions, practices, and experiences concerning pelvic and menstrual health and hygiene in India.

Stage 3: Selection of studies

Study selection followed a rigorous process to ensure that only studies fulfilling the predefined eligibility criteria were included. The inclusion criteria involved studies conducted on adolescents (girls) in India, articles published in the English language with full-text availability, research in which the outcomes assessed menstrual health and/or hygiene or puberty-related knowledge, published between 2011 and 2022, measured at least one of the outcomes on knowledge, attitude, and perceptions and source of information of pelvic health, puberty, menstrual health or hygiene, and were original research articles (quantitative, qualitative, or mixed-methods), reviews, or meta-analyses. Studies were excluded if they were conference abstracts, opinion pieces, editorials, non-peer-reviewed, or focused solely on sexual or emotional health without relevance to menstrual or pelvic health.

Moreover, the population, concept, and context (PCC) framework for the review was P = adolescent girls in India; C = knowledge, attitudes, and practices related to pelvic health, menstrual health, and hygiene; and C = Indian educational, social, and healthcare settings. Two reviewers independently screened titles and abstracts to remove duplicates and irrelevant studies. Full-text articles were then assessed for eligibility, with discrepancies resolved through discussion with a third reviewer.

Stage 4: Data charting

Data extracted from the included studies included study characteristics (authors, year, study design, location, and population), findings on KAP related to pelvic and menstrual health, sociocultural and environmental factors, educational gaps, and recommendations for improving awareness and practice. The data were organized thematically to identify key patterns and gaps.

Stage 5: Collating, summarizing, and reporting the results

The critical narrative technique was utilized to synthesize findings using text, tables, and figures to summarize and validate outcomes, along with thematic analysis. The results provided a comprehensive overview of the pelvic health, menstrual health, and hygiene scenario in India, highlighting sociocultural influences, educational and environmental barriers, and gaps in knowledge and practice. Recommendations were formulated to address these gaps and enhance awareness and education initiatives for adolescents in India.

Results

**Figure 1:**
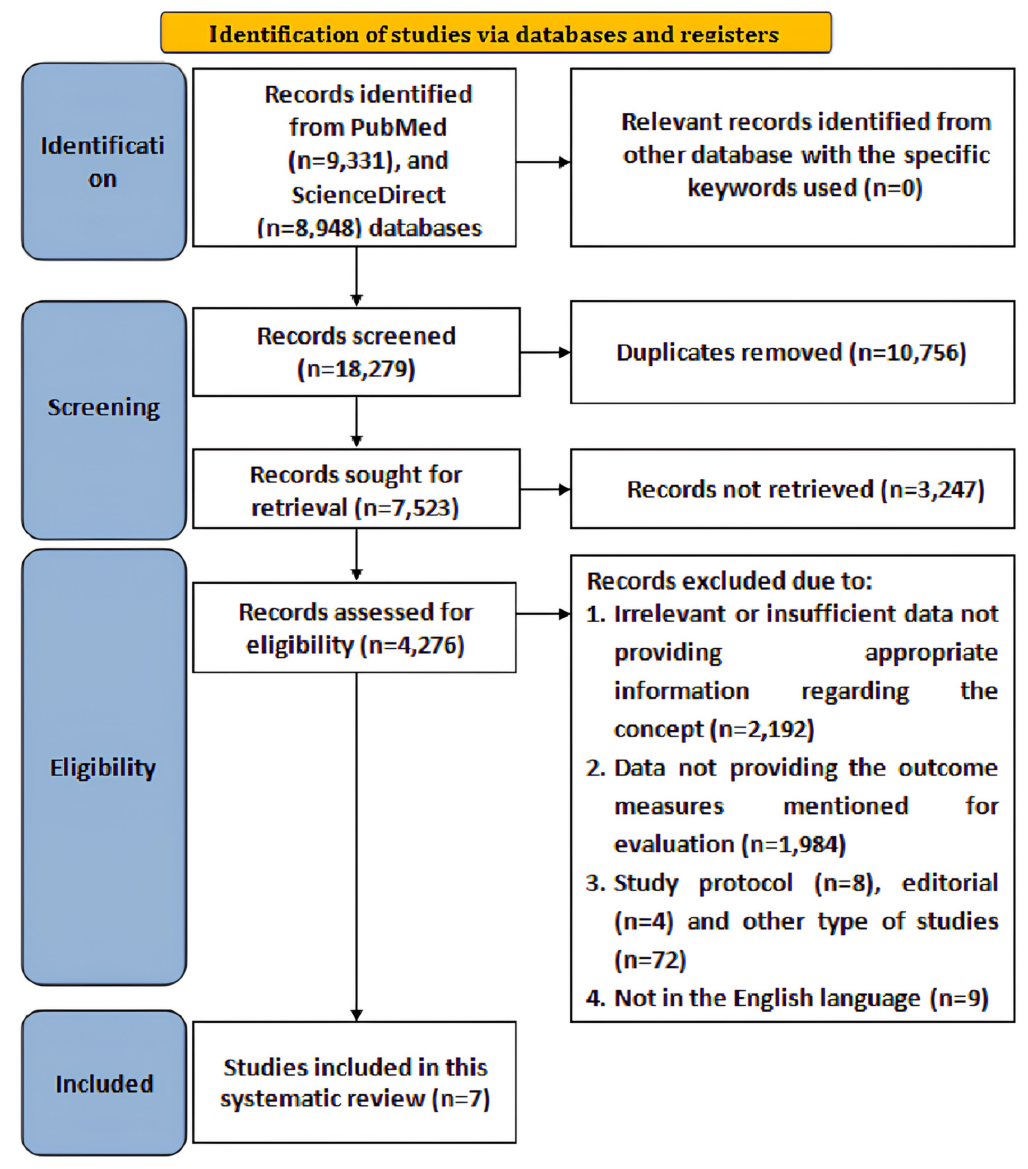
PRISMA flowchart PRISMA: Preferred Reporting Items for Systematic reviews and Meta-analyses

Initially, overall, 18,279 articles were found after searching the databases from 2011 to 2022 with the specified keywords. Duplicate articles consisting of 10,756 were removed after screening, after which 7,523 articles were sought for retrieval, of which 3,247 articles were not retrieved based on title and abstract evaluation. Moreover, overall, 4,276 records were assessed for eligibility from which 2,192 records were excluded due to irrelevant data not providing appropriate information regarding the concept (n = 2,192), data not providing the outcome measures mentioned for evaluation (n = 1,984), study protocol (n = 8), editorials (n = 4) and other types of studies (n = 72), and not in the English language (n = 9). Hence, seven studies fulfilling the eligibility criteria were included for the current review. The search strategy according to the Preferred Reporting Items for Systematic reviews and Meta-analyses (PRISMA) flowchart is illustrated in Figure 1.

The demographic characteristics of the included studies are reported in Table 1.

**Table 1:** Demographic details of the included studies LMICs: low- and middle-income countries; MHM: menstrual hygiene management; HSE: high socioeconomic; LSE: low socioeconomic

Author	Year	Study design	Study setting	Study population	Objective
Jena et al. [14]	2022	Descriptive cross-sectional study	Koraput district, Odisha, India	198 tribal adolescent schoolgirls	To explore the menstrual hygiene knowledge and practice among tribal students in Koraput, Odisha, India
Choudhary et al. [15]	2019	Cross-sectional, school-based comparative study	Urban and rural areas of Jodhpur district, Rajasthan, India	450 adolescent girls (235 urban and 215 rural)	To assess and compare the knowledge, perceptions, and practices of adolescent girls regarding menstrual hygiene in rural and urban areas of Jodhpur
Chandra-Mouli et al. [16]	2017	Systematic review	LMIC Including India	Adolescent girls are primarily school-going or mixed school-going/out-of-school, across urban, rural, and mixed settings	To determine knowledge among adolescent girls in LMIC about menstruation and how prepared are they for reaching menarche, to assess the sources of information regarding menstruation, to know how well do the adults around them respond to their information needs, and to evaluate the negative health and social effects the adolescents experience as a result of menstruation and their response when they experience these negative effects and the practices they execute as a result
Chothe et al. [17]	2014	Qualitative case study based on analysis of students' open-ended questions	Pune, Maharashtra, India	381 adolescent girls (62% response rate)	To document existing misconceptions regarding menstruation and perceptions about menarche and various menstrual restrictions that have been understudied
Phillips-Howard et al. [18]	2016	Narrative review and expert consensus outlining research priorities and methodologies for menstrual hygiene management	LMICs including India	Adolescent schoolgirls	Compile research priorities for MHM and types of research methods that can be used
van Eijk et al. [22]	2016	Systematic review and meta-analysis	India (includes urban, rural, slum, and mixed settings)	97,070 adolescent girls across 138 studies published from 2000 to 2015	To assess the status of MHM among adolescent girls in India to determine unmet needs
Jena et al. [23]	2017	Descriptive cross-sectional questionnaire-based study	Bhubaneswar, Odisha, India	600 adolescent school-going girls; 300 from an HSE group and 300 from an LSE group	To find out the prevalence of menstrual disorders and health-seeking behavior of adolescent school girls in two extreme socioeconomic groups

Moreover, the various findings on KAP related to pelvic and menstrual health, sociocultural and environmental factors, educational gaps, and recommendations for improving awareness and practice are reported in Table 2.

**Table 2:** Current scenario from evidences regarding knowledge, attitudes, and practices regarding pelvic health, menstrual health, and hygiene among adolescents in India WASH: poor water, sanitation, and hygiene; MHM: menstrual hygiene management; HSE: high socioeconomic; LSE: low socioeconomic; UNESCO: United Nations Educational, Scientific and Cultural Organization; UNICEF: United Nations International Children's Emergency Fund

Author and year	Findings on knowledge, attitudes, and practices related to pelvic and menstrual health	Psychological, sociocultural, and environmental factors associated	Educational gaps	Recommendations for improving awareness and practice
Jena et al. (2022) [14]	Knowledge: 81.8% had heard of menstruation premenarche, but none were fully informed; 57% had good knowledge; 80.8% knew menstruation’s physiologic basis, 39.9% identified the uterus as the blood source; 70.2% viewed blood as unhygienic. Main sources: friends (49.4%), mothers (39.9%). Attitudes: negative attitudes implied due to taboos; 6.6% saw menstruation as a curse, 48.5% misidentified blood source. Practices: 62% had normal cycles; 52% used cloth and pads, 34% pads only; 59% cleaned with soap/water, 35% water only; 63% changed absorbents 1–2 times/day; only 19% properly disposed; 29% had adequate MHM practices	Biological: poor hygiene increased infection risk. Psychological: shame and secrecy likely caused anxiety. Sociocultural: taboos and low parental education (68.2% mothers uneducated) limited accurate information. Environmental: limited pad access and facilities; 34% changed in cattle sheds, 3.7% in toilets	Incomplete premenarche knowledge; 43% lacked good knowledge; misconceptions prevalent; minimal input from teachers (5.1%) or health personnel (2.0%)	Increase awareness through education, provide affordable sanitary napkins via government programs, promote hygienic practices and disposal, address cultural taboos, and improve access to private facilities
Choudhary et al. (2019) [15]	Knowledge: 63.6% aware of menarche pre-onset; mothers' main information source (48.6%); only 21.8% knew of napkin subsidies (urban > rural, P rural, p = 0.026). Attitudes: 62.9% scared/depressed at first bleeding (rural > urban, p < 0.01); 40.7% viewed menstruation as impure; 23.3% supported restrictions. Practices: 97.3% bathed daily; 75.8% cleaned genitalia well (urban > rural, p < 0.01); urban girls used napkins more (56.2% vs. 29.3%, p < 0.01); 84.3% reused cloth; disposal varied (urban: dustbin, rural: burn, p < 0.01)	Biological: 92.7% had menstrual health issues (e.g., pain). Psychological: Fear/depression prevalent (rural > urban); shyness deterred doctor visits. Sociocultural: Taboos drove impurity views and restrictions (urban > rural, p < 0.01); low health-seeking (9.8% consulted doctors). Environmental: 96.4% of schools had toilets (74.7% clean); limited privacy/dustbins hindered pad-changing	Only 24.2% of girls had ever been counselled on menstrual hygiene. Awareness about adolescent health clinics was significantly higher among urban girls (25.1%) than rural girls (3.3%). Knowledge gaps and misconceptions were linked to inadequate school-based education and parental communication	Initiate regular menstrual hygiene awareness programs in schools, especially in rural areas. Incorporate menstrual health education into school curricula with trained teachers delivering content. Engage mothers and families in breaking myths and facilitating open conversations at home. Promote peer education models and involvement of community health workers, improve access to affordable and subsidized sanitary products, especially in rural settings. Ensure privacy, cleanliness, and disposal facilities in school toilets, strengthen links between adolescent girls and adolescent health clinics, and promote health-seeking behavior by reducing stigma around discussing menstrual problems with healthcare providers
Chandra-Mouli et al. (2017) [16]	Knowledge: awareness of menstruation before menarche varied widely (2.8% rural India to 100% urban Turkey); often inadequate (e.g., 75% of Chinese girls rated knowledge as inadequate). Misconceptions prevalent: 82% in rural Nepal viewed menstruation as a curse; only 6% saw it as physiological; 72.4% in India pre-education considered menstrual blood impure. Uterus as a bleeding source is known by ≤33% in India/Nepal, higher in Nigeria (78.7%), and Uganda (82.9%). Only 33% of rural Indian girls linked menarche to conception. Knowledge is higher among older girls, school-goers, and those with educated parents (p < 0.05). Attitudes: negative emotions dominated: 25–80% unprepared for menarche; fear, shock, disgust common (e.g., 30.5% Lebanon, 72.8% urban Malaysia felt blood was dirty). Pride in maturing reported by >50% in China, India, and Malaysia, but overshadowed by negative perceptions. Social stigma led to shame; Kenyan girls reported “hanging their head” due to unwanted attention. Religious restrictions were widespread (44.7-94.2% in India, 43.4% in Nigeria abstained from religious activities). Practices: Sanitary pad use varied: 2% rural Nepal to 93.8% urban Nigeria; higher in urban (e.g., 68.9% urban India) than rural (e.g., 11.3% rural India) settings (p < 0.01). Cloth use is prevalent in rural areas (e.g., 98% in Nepal, 69.2% in rural India); tissue/cotton is used in Uganda (37.1%). Daily bathing ranged from 0% (rural India) to 97.6% (urban India); urban higher than rural (p < 0.05). Absorbent changing at school is low (≤20% India/Egypt, 56.5% Nigeria) due to a lack of privacy/WASH facilities. Disposal methods: burning (17-76% India, 2.5% Egypt), burying, flushing; cloth reuse often unhygienic (30.7-72.4% dried in sunlight). School absenteeism: 2% in urban Nigeria to 61.7% in rural Uganda, linked to pain, dysmenorrhea, and lack of facilities	Biological: menstrual pain, cramps, fatigue, and dysmenorrhea caused absenteeism (p < 0.05 in Brazil, Lebanon). Poor hygiene practices (e.g., cloth reuse, inadequate genital cleaning) posed infection risks, though not directly evidenced. Psychological: fear, anxiety, sadness due to unpreparedness and stigma; 88.7% experienced negative changes postmenarche. Shame from leakage, fears, and societal taboos reduced confidence and school attendance. Sociocultural: taboos restricted activities (e.g., 87.4% of India avoided holy places, 28% of rural India limited household work). Mothers, primary informants (e.g., 94.4% India, 80% Malaysia), often shared misconceptions (e.g., menstruation as God’s will in Bangladesh). Cultural discomfort prevented teachers/parents from discussing menstruation (e.g., Kenyan male teachers, Tanzanian taboos). Environmental: Inadequate WASH facilities: insufficient latrines, water, and disposal systems in schools (India, Tanzania, Uganda). Cost barriers drove cloth use in rural areas (e.g., Kenyan girls used cloth to save money). Lack of privacy at school/home hindered absorbent changing and bathing (e.g., Kenyan girls feared revealing menstruation)	Limited premenarche awareness (e.g., 2.8-100% aware); only 6-96.7% understood menstruation as physiological. Schools rarely provided MHM education; teachers cited as sources by <5% in India, Nepal, and Sri Lanka. Mothers, sisters, and friends are dominant sources (e.g., 92.2% Egypt cited mothers), but often untimely/inaccurate. Health professionals rarely consulted (<1% India, Jordan); mass media supplementary but inconsistent (29.2-92.2%). Lack of standardized MHM curricula; girls in Egypt, Nepal sought more information but felt ashamed asking	Implement multilevel interventions: educate girls/boys on puberty via school-based programs (e.g., India, Egypt, Iran showed improved knowledge/practices, p < 0.01), and encourage parental communication (e.g., Families Matter Program). Improve access to sanitary pads, water, toilets, and privacy; promote safe disposal. Train health workers for competent MHM care. Shift cultural norms to view menstruation as normal, not shameful, via community campaigns (e.g., Procter and Gamble’s programs). Scale up initiatives like UNESCO’s policy booklet, UNICEF’s WASH in schools, and Columbia/Save the Children’s puberty booklets. Conduct rigorous research with standardized MHM measures to address data gaps and cultural variations
Chothe et al. (2014) [17]	Knowledge: limited understanding of menstruation and reproductive anatomy; primary information from mothers, often inaccurate. Attitudes: negative, driven by myths (e.g., impurity) and taboos, causing fear and embarrassment. Practices: poor menstrual hygiene due to inadequate knowledge of sanitary pad use; reliance on cloth pieces and home remedies over medical advice	Sociocultural: taboos restricted activities (e.g., temple visits); myths (e.g., infertility from pad disposal) perpetuated by family norms. Environmental: lack of school water, bathrooms, and pad disposal facilities hindered hygiene	No formal menstrual health curriculum; untrained teachers; excluded topics like abnormalities and contraception; inadequate premenarche education. Moreover, boys were often excluded from related sessions, contributing to gender-based knowledge disparities and social teasing	Implement grade-specific sex education with visual aids, train teachers/parents for counselling, improve school sanitation facilities, promote low-cost sanitary pads and community health services, conduct further research for culturally sensitive interventions, and necessary implementation of policy
Phillips-Howard et al. (2016) [18]	Knowledge: limited MHM guidance; poor understanding of menstrual cycle-fertility links. Attitudes: taboos cause shame, fear, and low self-esteem, driven by gender inequality. Practices: use of unhygienic materials, transactional sex for pads, and poor school facilities hinder hygiene	Biological: risk of infections from poor MHM; needs lab confirmation. Psychological: stress, anxiety, and low confidence from a lack of support. Sociocultural: gender norms, taboos, and male influence limit MHM; cultural barriers to product use. Environmental: WASH in school and disposal facilities	Lack of standardized MHM curricula, outcome measures, educator training, and support for vulnerable groups like disabled girls or those out of school	Conduct multisite trials, longitudinal studies, and reviews to quantify MHM impacts, improve WASH facilities, MHM product access, and school education; support vulnerable girls, establish MHM research consortium, standardize measures, increase funding, and translate evidence into policy
van Eijk et al. (2016) [22]	Knowledge: 48% aware of menarche pre-onset (urban 53%, rural 45%); mothers' primary source (52%); 23% knew uterus as bleeding source; 55% viewed menstruation as normal. Attitudes: stress and shame are common; 77% faced religious restrictions; 38% faced food restrictions. Practices: urban pad use, 67%; rural, 32%; rural cloth use, 63%; 23% improper disposal; 84% bathed daily; 37% changed pads at school; 24% missed school	Biological: pain drove absenteeism; poor hygiene risked infections (e.g., cloth reuse without proper cleaning, disposal in open spaces). Psychological: menarche caused anxiety; fear of staining deterred attendance. Sociocultural: taboos (77% religious restrictions); mothers’ misinformation; virginity concerns, limited cup use. Environmental: 51% had home toilets; poor school WASH hindered pad-changing; cost drove cloth use	Only 48% were aware of menarche beforehand, and just 23% understood its physiological basis, indicating inadequate puberty education. Schools rarely provided MHM education; teachers were minor information sources (8-12%). Reliance on mothers (54%) and friends (24%) for knowledge highlighted the absence of formal curricula. Low awareness of hygienic absorbents (e.g., pads, menstrual cups) and proper disposal methods, especially in rural/slum settings. Lack of data on school sanitation facilities limited analysis of WASH impacts on MHM practices	Strengthen MHM programs through: enhanced puberty education in schools, including age-appropriate sex and relationship curricula to teach physiology and dispel myths, and training mothers and community health workers to provide practical MHM knowledge. Improve access to hygienic absorbents (e.g., affordable pads, menstrual cups) and promote their use, particularly in rural areas. Address disposal challenges by developing eco-friendly pads and safe disposal systems to manage increasing waste (e.g., 9000 tons annually). Upgrade school WASH facilities (separate toilets, water, and disposal systems) to support MHM and reduce absenteeism. Implement cultural and societal interventions to challenge taboos and reduce restrictions, fostering positive attitudes toward menstruation. Conduct higher-quality studies with standardized MHM definitions and rigorous methods to better inform policy
Jena et al. (2017) [23]	Knowledge: only 65% of HSE vs. 10.1% LSE found reproductive health knowledge adequate; 89.9% LSE felt deficient. Mothers (75% HSE) and school (58.23% LSE) were primary sources; 73% HSE, 80.1% LSE were unaware of LMP (p = 0.039). Attitudes: 55% HSE, 47.8% LSE viewed menstruation as normal; negative emotions included disgust (26.7% HSE), fear (25.81% LSE); 41% HSE, 20.9% LSE embarrassed by reproductive health questions. Practices: regular cycles in 64% HSE, 76.09% LSE (p = 0.001); scanty flow in 69.69% LSE, average in 62% HSE (p < 0.00001). Dysmenorrhea affected 72% of HSE, 52% of LSE (p = 0.0036). School absenteeism: 17% HSE, 9.76% LSE; advice sought by 38% HSE, 13.45% LSE	Biological: earlier menarche in HSE (11.8 vs. 12.7 years LSE) linked to obesity; scanty flow in LSE tied to malnutrition. Psychological: fear, confusion, and embarrassment hindered help-seeking. Sociocultural: LSE relied on less-educated mothers and friends; reluctance to consult male doctors. Environmental: LSE’s underprivileged setting has limited facilities and knowledge	High knowledge deficits in LSE (89.9%), minimal STD/contraception awareness, and low LMP tracking; cultural taboos stifled discussion	Educate girls and mothers, integrate menstrual health into school curricula, promote mother-girl programs, address consultation barriers (e.g., male doctors, embarrassment), improve access to female providers, and research sociocultural barriers in underprivileged groups

Thematic analysis

Myths and Taboos Surrounding Menstruation

Deep-rooted myths and taboos foster negative perceptions and sociocultural restrictions around menstruation, particularly in India. Comparative analysis from India showed higher negative attitudes toward menarche in rural compared to urban areas, but both regions had a significant spread of taboos associated with menstrual hygiene. Moreover, social nonacceptance in discussing menarche and puberty has been prevalent in the country [14-18,22,23]. The myth of impurity and nonparticipation in religious customs and gatherings persists in most. Certain restrictions in basic activities like entering the kitchen, cooking, and serving food during menstruation are still very common. Isolation in cooking and eating separately, staying in separate rooms, and sleeping on separate beds is due to the misconception of impurity and untouchability. In most communities, lack of initiative in education results in compromised menstrual hygiene practices due to limited awareness [14-18,22,23].

Access to Knowledge and Support (Pre- and Postmenarche)

Societal taboos and scarce educational resources contribute to a limited understanding of menstruation among adolescent girls. Studies consistently identify mothers as the primary source of menstrual information, yet their guidance is often incomplete or inaccurate. School-based education on menstruation lacks standardization, varies widely in delivery and content, and is frequently insufficient. Notably, boys are excluded from these educational efforts, leaving them without biological knowledge and reliant on unreliable sources such as the media and the internet. Other information sources for girls include friends, teachers, and online platforms, though these are also limited in reliability. There is an urgent need for comprehensive, age-appropriate educational modules to address the physical and emotional aspects of puberty and menstruation. Workshops engaging healthcare professionals, educators, and community leaders are recommended to enhance knowledge access. Creating an open, supportive environment is critical to empower adolescents to manage their pelvic and menstrual health effectively. A multifaceted approach combining educational initiatives can improve healthcare access, dispel myths, and overcome cultural barriers, fostering holistic well-being [14-18,22,23].

Sources of Information: Curriculum Lacunae and Lack of Teachers’ Training Standards

Schools lack standardized training programs for teachers to deliver education on pelvic health, menstrual health, and hygiene, resulting in inconsistent and incomplete instruction. The existing curriculum provides only minimal information, focusing solely on the female reproductive system and the monthly menstrual cycle, while omitting critical details about pelvic organs, their anatomy and function, associated myths, and essential menstrual hygiene practices. This narrow, biology-centric approach neglects the physical and mental health implications of menstruation and fails to address hygiene needs. Furthermore, male students are consistently excluded from menstrual health sessions due to teachers’ lack of training to navigate embarrassment and confidently engage both genders in these discussions. Pelvic health remains largely overlooked, compounded by the absence of expertise among educators conducting menstrual awareness programs [14-18,22,23].

Environmental Factors: Water, Sanitation, and Resource Availability

Studies highlight that adolescents face significant obstacles in maintaining menstrual hygiene due to limited access to resources and environmental constraints. Factors such as water availability, sanitation infrastructure, and sanitary products critically affect effective hygiene practices. Water shortages in schools hinder safe and comfortable menstrual management, while inadequate disposal facilities lead to unhygienic practices. Overcoming these environmental barriers requires a comprehensive strategy, including education, stakeholder engagement, and policy advocacy, to create supportive environments that promote menstrual and pelvic health and well-being [14-18,22,23].

Lack of Knowledge and Beliefs Leads to Altered/Inadequate Coping Mechanisms and Behavioral Adaptations

Research indicates a strong link between limited menstrual knowledge and negative attitudes toward health and hygiene. Insufficient school-based education fails to equip adolescents with the behavioural strategies needed to manage emotions such as embarrassment, fear, and shame triggered by menarche. Providing inclusive education for both boys and girls can mitigate stigma and promote empathy. Lack of understanding about pelvic and menstrual health, coupled with poor hygiene knowledge, creates significant obstacles to effective education and self-care, while inadequate coping mechanisms intensify these emotional challenges [14-18,22,23].

Biological Differences and Consequences

Studies link higher school absenteeism among adolescent girls to an insufficient understanding of menstruation and poor management of related discomfort. Limited awareness of menstrual product options and the importance of hygiene during periods contributes to negative outcomes, including difficulty concentrating and increased fatigue [14-18,22,23].

MHM Standards

Government efforts in MHM fall short: The Indian government has introduced initiatives to improve MHM, notably the Swachh Bharat Mission launched in 2014, which seeks to enhance community sanitation facilities to support hygiene and menstrual health. Despite these efforts, inconsistent standards and limited access to necessary resources hinder effective sanitation and hygiene outcomes. While school-based awareness campaigns and camps are organized, they lack a uniform approach to delivering knowledge and practical guidelines, undermining their impact [14-18,22,23].

Gender Differences and Cultural Gaps

School systems fail to provide equitable and comprehensive menstrual health education to both boys and girls, contributing to persistent taboos and teasing. This gap underscores the need for culturally sensitive, inclusive education programs that address both genders [14-18,22,23]. Societal norms, cultural practices, and inadequate educational approaches have not closed the knowledge and attitude divide, particularly among boys, regarding menstrual and pelvic health. Pelvic health, a critical preventive approach, remains unevenly taught, with boys largely excluded. Despite their potential as future policymakers and key stakeholders in supporting girls’ menstrual hygiene, boys lack access and engagement in this education. Cultural norms that stifle open dialogue between genders further limit awareness and understanding, reinforcing stigma and hindering progress.

Discussion

This scoping review synthesizes evidence on the KAP related to pelvic and menstrual health among adolescent girls in India, applying the biopsychosocial model to reveal eight key themes: myths and taboos, access to knowledge, sources of information, environmental factors, coping mechanisms, biological consequences, MHM standards, and gender disparities. The findings from the seven included studies highlight persistent sociocultural, educational, and environmental barriers that undermine adolescent menstrual health, aligning with the biopsychosocial model’s emphasis on biological, psychological, and social influences [14-18,22,23]. Though pelvic health is integral to the review’s scope, the limited focus on pelvic health outcomes in the included studies reflects a research gap, as most prioritize menstrual health, highlighting the need for future studies on pelvic floor disorders and related conditions in adolescents.

Studies by Chothe et al., Choudhary et al., Chandra-Mouli et al., and Jena et al. underscore the pervasive influence of myths, such as menstrual blood being impure or a curse, leading to restrictions like avoiding religious activities or household tasks [14-17]. These findings echo earlier research by Garg and Anand, who noted that 70-90% of Indian girls face cultural restrictions during menstruation, reinforcing social stigma [24]. All studies highlighted limited pre- and postmenarche knowledge, with mothers as the primary, yet often inaccurate, information source [14-18,22,23]. Jena et al. found that none of the tribal girls had complete knowledge despite 81.8% awareness, mirroring van Eijk et al., where only 23% understood the uterus as the bleeding source [14,22]. This aligns with Sommer et al., who noted that reliance on informal sources in LMICs perpetuates misconceptions due to inadequate formal education [25]. The exclusion of boys from education, as noted by Chandra-Mouli et al., parallels findings by Mason et al., where boys’ lack of menstrual knowledge contributed to teasing and stigma [16,26].

The absence of standardized teacher training and comprehensive curricula was evident across studies [14-18,22,23]. Chothe et al. and Phillips-Howard et al. reported biology-focused curricula neglecting hygiene and psychosocial aspects, consistent with Sivakami et al., who criticized Indian school curricula for omitting practical MHM guidance [17,18,27]. Teacher discomfort and male student exclusion, noted by Chandra-Mouli et al., align with Shah et al., who found untrained teachers avoided menstrual discussions due to cultural embarrassment [16,28]. Poor water, sanitation, and hygiene (WASH) facilities were significant barriers, as reported by van Eijk et al. and Jena et al., with only 3.7% of tribal girls using toilets for absorbent changing [14,22]. These findings corroborate Sommer et al., who linked inadequate school WASH to reduced MHM practices in LMICs [25]. Cost-driven cloth use, noted by Chandra-Mouli et al., aligns with Thakur et al., where 60% of rural Indian girls used cloth due to pad inaccessibility [16,29].

Limited knowledge correlated with negative emotions (e.g., fear, shame), as seen in Choudhary et al. and Jena et al., where 62.9% and 41% of girls, respectively, felt scared or embarrassed [15,23]. This is consistent with McMahon et al., who reported anxiety and secrecy among Indian girls due to inadequate preparation [30]. Inclusive education, recommended by Phillips-Howard et al., could reduce stigma, as supported by Hennegan et al., who found gender-inclusive programs fostered empathy [18,31]. School absenteeism linked to dysmenorrhea and poor hygiene knowledge was reported by van Eijk et al. and Jena et al., with 24% and 17% absenteeism rates, respectively [22,23]. These findings align with Vashisht et al., who noted 20-40% absenteeism among Indian girls due to menstrual discomfort and lack of facilities [32]. Fatigue and concentration issues, as per Chandra-Mouli et al., corroborate Grant et al., linking poor MHM to academic underperformance [16,33].

Government initiatives like the Swachh Bharat Mission were critiqued for inconsistent implementation by Jena et al. and Phillips-Howard et al. [16,23]. Successful MHM programs in Kenya, as per Phillips-Howard et al., suggest that India could benefit from standardized guidelines and better resource allocation [18]. The exclusion of boys from menstrual education, noted by Chothe et al. and Chandra-Mouli et al., perpetuated teasing and taboos [16,17]. This is consistent with Mahon and Fernandes, who emphasized the need for male inclusion to shift cultural norms [34]. Culturally adapted education, as recommended by Choudhary et al., aligns with Sommer et al., who advocated for community-based interventions to address gender disparities [15,35].

Comparatively, the review’s findings highlight India’s unique challenges, such as deep-rooted cultural taboos and resource constraints, which are more pronounced than in other LMICs like Nigeria or Kenya, where pad use and WASH facilities are relatively better [7,16]. However, India’s government initiatives show progress, though less effective than global models like the United Nations Educational, Scientific, and Cultural Organization (UNESCO’s) puberty education programs [7]. The biopsychosocial framework applied here provides a novel lens, integrating biological (e.g., infection risks), psychological (e.g., shame), and social (e.g., taboos) factors, unlike prior reviews that focused solely on MHM [6,18].

Limitations

This scoping review has several limitations. First, the inclusion of studies from 2011 to 2022 may limit the inclusion of recent developments, though this timeframe aligns with the review’s scope. Second, the focus on full-text English-language articles may exclude relevant studies, potentially limiting cultural perspectives. Third, the heterogeneity of study designs (qualitative, quantitative, reviews) and settings (urban, rural, and tribal) complicates direct comparisons.

Recommendations for future research

Conduction of longitudinal studies to assess the long-term impact of menstrual health education on health outcomes, evaluation of the effectiveness of gender-inclusive, culturally adapted education programs, development and validation standardized outcome measures for MHM, investigation regarding the impact of improved WASH facilities on MHM practices and school attendance, and conduction of research studies in tribal and rural settings to address unique sociocultural and environmental barriers. Moreover, exploration of the efficacy of digital tools (e.g., apps, online modules) for delivering menstrual health education, given increasing internet access in India, should be focused. Additionally, assessment of the barriers for implementation of the government MHM initiatives, to inform scalable solutions [6].

## Conclusions

This scoping review provides a comprehensive overview of the KAP surrounding pelvic health, menstrual health, and hygiene among adolescents in India. The findings underscore the pervasive influence of cultural taboos, inadequate education, and environmental constraints, which contribute to poor MHM, psychosocial distress, and educational disruptions. By applying the biopsychosocial model, the review highlights the interplay of biological, psychological, and social factors, offering a holistic perspective absent in prior reviews. While government initiatives show promise, their inconsistent implementation limits impact. Addressing these challenges requires standardized, inclusive education, improved facilities, and culturally sensitive interventions to promote adolescent well-being, ultimately fostering a healthier, more equitable society.
